# Product Management Experiences of Patients With a Stoma Receiving Telenursing Services

**DOI:** 10.1002/nop2.70755

**Published:** 2026-10-19

**Authors:** Evin Korkmaz, Gökçe Akkaya, Kübra Can, Seçil Taylan, Fatma Eti Aslan

**Affiliations:** ^1^ Independent Researcher Izmir Türkiye; ^2^ Bahcesehir University Graduate School of Education (PhD Student) Istanbul Türkiye; ^3^ Department of Nursing, Faculty of Health Sciences Haliç University Istanbul Türkiye; ^4^ Department of Nursing, Department of Surgical Nursing, Faculty of Health Sciences Akdeniz University Antalya Türkiye; ^5^ Department of Nursing, Faculty of Health Sciences Bahcesehir University Istanbul Türkiye

**Keywords:** product management, qualitative study, stoma care, telehealth, telenursing

## Abstract

**Aim:**

This study explored how individuals with a stoma interpret and manage product‐related challenges and examined the contribution of telenursing services to their daily care processes.

**Design:**

A qualitative descriptive design.

**Methods:**

Seven participants were recruited through criterion‐based purposive sampling among individuals receiving telenursing support for stoma care from a private healthcare provider. Data were collected through online semi‐structured interviews lasting 20–30 min and analysed using conventional content analysis with MAXQDA 24 software. The study was reported according to COREQ guidelines. Ethical approval was obtained from the Bahçeşehir University Scientific Research and Publication Ethics Committee prior to data collection (decision no. 2024/07, dated 1 August 2024). All participants provided written informed consent, and institutional approval was obtained before the study commenced.

**Results:**

Five overarching themes were identified: reasons for using telehealth, recommendations received through telenursing, emotional responses following telehealth, perceived effectiveness of counselling and alternative methods sought by patients. Participants reported that telenursing provided practical, timely and personalised guidance that helped them manage issues such as adapter leakage, skin irritation, product selection and wound assessment. Participants reported needing fewer hospital visits and described feelings of safety, satisfaction and relief. While some participants consulted physicians or the internet for additional information, telenursing remained their primary and trusted source of support.

**Conclusion:**

Participants described telenursing as supporting their self‐care capacity and as a source of practical guidance and reassurance in managing product‐related problems. These accounts suggest that integrating telehealth into routine stoma care may strengthen continuity of care, although the present design does not allow its effects on clinical outcomes to be determined. Health professionals should consider expanding digital care competencies and developing structured telenursing protocols to strengthen this form of support.

**Implications for the Profession and/or Patient Care:**

Participants' accounts suggest that structured telenursing support may help individuals with a stoma manage practical, product‐related difficulties in daily life. Stoma nurses and telenurses can use these findings to inform product‐use education, routine skin assessment, leakage management, structured follow‐up contacts, timely escalation to physicians and the development of digital communication protocols.

**Impact:**

What problem did the study address? Individuals living with a stoma frequently encounter difficulties with product selection, adapter fit, leakage and peristomal skin care, yet little is known about how these difficulties are experienced in everyday life, or how telenursing responds to them from the patients' own perspective. What were the main findings? Participants' accounts centred on practical product problems such as adapter leakage, peristomal irritation and wound care. They described the remote guidance they received as prompt and dependable, said it left them feeling more secure and better able to manage their own care, and reported turning to hospital services less often than they otherwise would have. Other information sources were consulted, but telenursing was consistently placed ahead of them. Where and on whom will the research have an impact? The findings are relevant to stoma care nurses and telenurses, and to the healthcare organisations that provide remote follow‐up for individuals living with a stoma.

**Reporting Method:**

The study adhered to the EQUATOR Network guidelines and was reported in accordance with the Consolidated Criteria for Reporting Qualitative Research (COREQ) checklist.

**Patient or Public Contribution:**

Participants contributed to the study by sharing their experiences in individual interviews and by reviewing summaries of their own interviews as part of member checking, which informed the verification of the interpretations reported here. Patients or members of the public were not involved in the design of the study, in the development of the interview guide, or in the preparation of the manuscript.

## 1 Introduction

A stoma is an artificial opening created by suturing a segment of the small or large intestine to the abdominal wall as a medical necessity (Öztürk and Karadağ 2019). An intestinal stoma (colostomy or ileostomy) is often performed to sustain life, improve quality of life and support the management of the underlying disease (Duluklu and Çelik 2019). Although stoma creation is a life‐saving intervention, it may lead to complications and negatively affect individuals' physical, emotional and social well‐being (Incesu and Ulupınar 2022). Individuals living with a stoma frequently experience body image disturbance, limitations in daily activities and challenges related to sexuality (Taylan and Akıl 2020). Stoma nurses play a key role in evaluating patients' care needs, guiding product selection and supporting individuals in becoming independent in their stoma care (Öztürk and Karadağ 2019; Incesu and Ulupınar 2022; Taylan and Akıl 2020; Baykara et al. 2022).

With the increasing digitalisation of healthcare, telenursing has gained importance as a complementary model of care. Telehealth has been defined as the use of communication technologies to support and improve patient care (Sevinç and Aktürk 2024). It enables individuals to access professional support remotely and offers significant advantages, especially for those with chronic conditions or requiring specialised postoperative care (Kim et al. 2023; Koçan et al. 2023; Sun et al. 2020). Patients with a stoma often face ongoing challenges related to stoma care and product management and therefore require continuous guidance from healthcare professionals (Sun et al. 2020; Köstekli et al. 2020). Evidence indicates that telehealth support regarding product selection, maintaining skin integrity and preventing complications enhances patients' experiences and quality of life (Stavropoulou et al. 2021; Taylan and Aksoy 2021). Furthermore, difficulties in using and maintaining stoma products are particularly common after cancer treatment, highlighting the need for constantly improving product technology and design (Sevinç and Aktürk 2024).

Previous studies have shown that telehealth contributes positively to stoma adaptation, self‐efficacy and satisfaction and reduces stoma‐related complications during the postoperative period (Sevinç and Aktürk 2024; Taylan and Aksoy 2021; Wei‐ying et al. 2024). Research has also addressed the product selection process for individuals with a stoma (Harris 2021; Tsujinaka et al. 2020). However, the number of studies focusing specifically on the difficulties experienced in stoma product management and the role of telehealth in addressing these challenges remains limited. In particular, little is known about how individuals with a stoma experience product‐related challenges in their daily lives and how telenursing responds to these challenges from the patients' own perspective.

Therefore, this study explores the product management experiences of individuals living with a stoma and the meanings they attribute to these experiences. It also aims to elucidate how telehealth services contribute to this process from the perspective of stoma product users.

## 2 Materials and Methods

### 2.1 Study Design

This study employed a qualitative descriptive research design to explore how individuals with a stoma manage stoma‐related products and interpret the telenursing support they receive. This approach enabled an in‐depth exploration of participants' experiences and perspectives regarding product management and telenursing support.

### 2.2 Study Population and Sampling Strategy

The study was conducted with individuals who received telenursing support for stoma care from a private healthcare provider. Telenursing was not delivered as a stand‐alone intervention; it was a standard component of the routine follow‐up service that the provider offers to all individuals using its stoma‐care products. Participants did not pay any additional fee for this service. A criterion‐based purposive sampling strategy was used to recruit participants who met the predetermined eligibility criteria. Recruitment continued until data saturation was reached, which was defined as the point at which no new codes or concepts emerged from consecutive interviews.

### 2.3 Inclusion Criteria

Participants were eligible if they:

- voluntarily agreed to participate;
- were able to communicate in Turkish;
- were 18 years of age or older;
- had no speech or hearing impairments;
- had an intestinal stoma (colostomy or ileostomy);
- were at least 15 days post‐surgery, discharged from hospital and reintegrated into daily life;
- received telenursing services from the study institution.

Individuals who did not meet these criteria were excluded.

### 2.4 Data Collection

Data were collected between 30 September and 30 October 2024 through single‐session, semi‐structured interviews lasting approximately 20–30 min, conducted via an online videoconferencing platform. A descriptive information form and a semi‐structured interview guide, developed by qualitative research experts and informed by the literature (Creswell and Poth 2016; Patton 2014), were used.

To enrich the narratives, probing questions such as ‘Could you describe this further?’, ‘How did this situation make you feel?’ and ‘What did this experience mean to you?’ were used. All interviews were audio‐recorded with permission and transcribed verbatim. Data saturation was reached at the sixth interview, at which point no new codes or concepts emerged from the data; a seventh interview was conducted to confirm saturation, and no additional codes were identified. Thus, the final sample comprised seven participants.

All interviews were conducted by the second author (G.A.), a nurse who provides consultancy as a telenurse for the healthcare provider and who had previously delivered telenursing support to the participants. This prior relationship facilitated rapport and the detailed recall of specific care episodes, but it also carried a risk of social desirability bias. To reduce this risk, the interviews were arranged as separate appointments, distinct from the routine telenursing service the participants received, and were conducted only with individuals who had agreed to take part in the study. Participants were informed that the service they received would not be affected by the feedback they provided, that the data would be shared only with the researchers involved in the study and that their identifying information would be kept confidential. In addition, the coding and interpretation were reviewed independently by two qualitative researchers, and participants were provided with summaries of their interviews to confirm or correct the interpretations.

### 2.5 Data Analysis

Data analysis consisted of two stages. First, a descriptive analysis summarised the participants' sociodemographic characteristics. Next, a conventional content analysis approach was used to analyse the interview transcripts. Coding was conducted using MAXQDA 24 software (VERBI Software, Berlin, Germany). Meaning units were identified, condensed and grouped into codes, which were then organised into categories and overarching themes. Discrepancies between researchers were resolved through discussion until consensus was reached. Reporting followed the Consolidated Criteria for Reporting Qualitative Research (COREQ).

### 2.6 Trustworthiness

Several strategies were adopted to ensure the trustworthiness of the study.

#### 2.6.1 Credibility

The analysis was independently reviewed by two qualitative research experts.

#### 2.6.2 Member Checking

Participants were provided with summaries of their interviews to verify the accuracy of interpretations.

#### 2.6.3 Confirmability

Direct quotations were included to reflect the participants' experiences authentically.

#### 2.6.4 Dependability

An audit trail documenting coding decisions and analytical procedures was maintained to ensure methodological transparency.

#### 2.6.5 Transferability

A thick description of the participants, the service context and the study setting was provided so that readers may assess the applicability of the findings to other settings. Table 1 presents each participant's clinical and experiential characteristics, including age, diagnosis, stoma type, surgical urgency, appliance type and the source of initial stoma‐care education. The nature of the telenursing service, its position within the provider's routine follow‐up and the absence of any additional cost to participants are described in Section 2.2, while the interview period, format and duration are reported in Section 2.4.

**TABLE 1:** Individual clinical and experience characteristics of participants.

Participant	Age	Gender	Education	Reason for surgery	Surgery type	Education source	Product used
Participant 1	70	Female	High School	Cancer	Emergency	Company Training	Convex Adapter
Participant 2	70	Male	High School	Cancer	Emergency	Company Training	One‐piece Product
Participant 3	77	Male	High School	Cancer	Emergency	Company Training	Flat Adapter
Participant 4	61	Female	University	Cancer	Planned	Stoma Nurse	Convex Adapter
Participant 5	43	Male	High School	Cancer	Emergency	Company Training	Convex Adapter
Participant 6	25	Male	Primary School	Perforation	Planned	Stoma Nurse	Convex Adapter
Participant 7	18	Male	Illiterate	Ulcerative Colitis	Emergency	Company Training	Two‐piece Product

*Note:* The table summarises demographic variables, clinical characteristics, training processes, and stoma‐related experiences of the participants included in the study.

### 2.7 Ethical Considerations

Ethical approval for the study was obtained from the Bahçeşehir University Scientific Research and Publication Ethics Committee (decision no. 2024/07, dated 1 August 2024). Written permission was obtained from the healthcare institution where the study was conducted. All participants provided written informed consent.

## 3 Findings

The seven participants (P1–P7) ranged in age from 18 to 77 years, the majority were male, and most had undergone emergency surgery due to cancer. For most participants, this was their first experience with a stoma, and their initial stoma‐care education had been provided either by company‐based trainers or a stoma nurse. Participants used various types of stoma appliances, including flat, convex, one‐piece and two‐piece systems.

Content analysis yielded five overarching themes, each comprising several subthemes. These themes reflect the lived experiences of individuals with a stoma as they navigate challenges related to product management and the ways in which telenursing shapes their care practices, emotional responses and decision‐making processes. The code cloud illustrating the relationships among the themes is presented in Figure 1.

**FIGURE 1:**
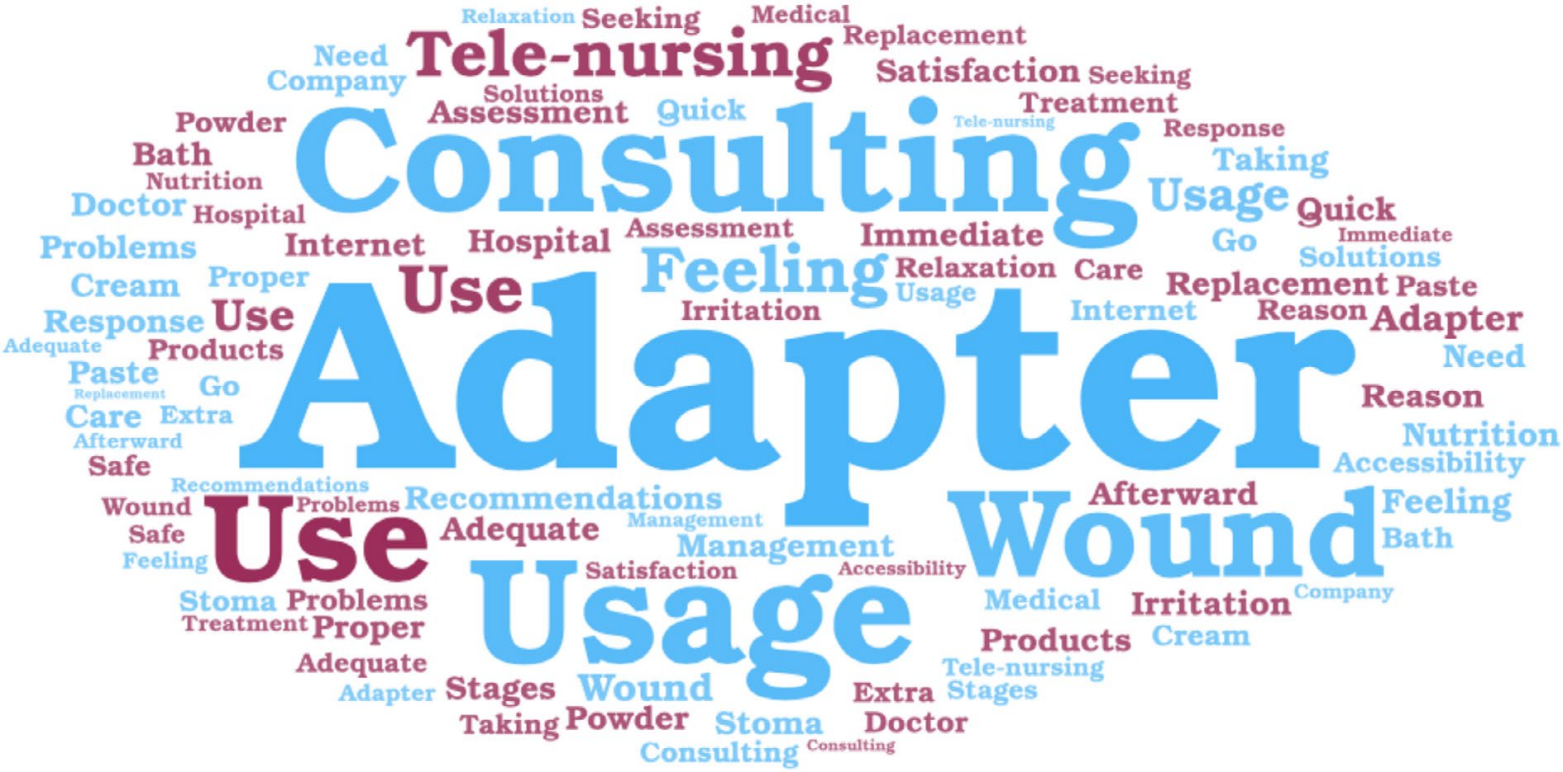
Code cloud illustrating key concepts related to stoma product‐management experiences among patients receiving telehealth services.

### 3.1 Theme 1: Reasons for Using Telehealth

Participants described multiple reasons for seeking telenursing support during their stoma‐care process. These reasons were grouped into five subthemes, which highlight the specific challenges experienced in daily stoma management (Figure 2).

**FIGURE 2:**
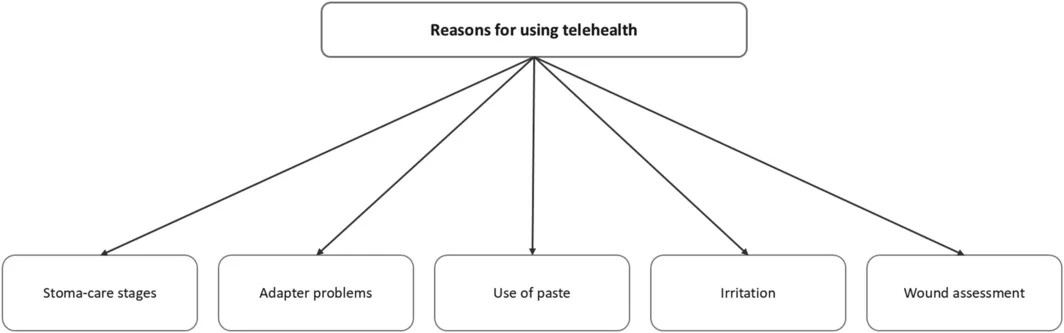
Reasons for using telehealth, presented with corresponding sub‐themes derived from participants' narratives.

#### 3.1.1 Subtheme 1: Stoma‐Care Stages

Participants often relied on telehealth to clarify uncertainties in routine stoma‐care procedures. Guidance was particularly needed in determining the appropriate timing for cleaning and managing bodily changes.

At first, I had a lot of trouble knowing when to clean it. We used to do it in the afternoon … my stomach was full, there was activity and bleeding. After talking to you, we realised it was much easier on an empty stomach. (P5)

#### 3.1.2 Subtheme 2: Adapter Problems

Many participants described persistent leakage, improper fit or irritation caused by unsuitable adapters. Telehealth consultations helped them identify alternative products and make timely adjustments.

Our adapter was flat at first… there was constant leaking. We sent pictures, and you recommended a dimpled adapter. After that, the leakage stopped. (P4)

The non‐pitted adapter irritated my stoma. After learning from you, I tried a dimpled one, and it was much more comfortable. (P3)

#### 3.1.3 Subtheme 3: Use of Paste

Participants noted that telenursing support helped them better understand how to use skin protectors and pastes effectively.

I learned how to use the pastes properly… It was very useful, and I'm glad it happened. (P3)

#### 3.1.4 Subtheme 4: Irritation

Irritation around the stoma was a common concern. Telehealth guidance helped participants recognise causes and receive product recommendations.

There were sores, and you suggested a non‐allergenic product. (P5)

There were cracks and bleeding. You recommended creams, and they helped a lot. (P3)

#### 3.1.5 Subtheme 5: Wound Assessment

Participants frequently sought assistance when evaluating wound appearance and determining appropriate interventions.

You guided us on what product to use and how to apply it. (P2)

I would send photos of the wounds, and you would tell me what to do. (P6)

Summary of Theme 1:

Overall, participants used telehealth primarily to address uncertainties in product use, manage complications and ensure safety and accuracy in daily stoma‐care routines.

### 3.2 Theme 2: Recommendations Received Through Telehealth

This theme illustrates the diverse range of recommendations participants received during telenursing consultations, extending beyond product use to encompass hygiene, nutrition, medical guidance and wound care (Figure 3).

**FIGURE 3:**
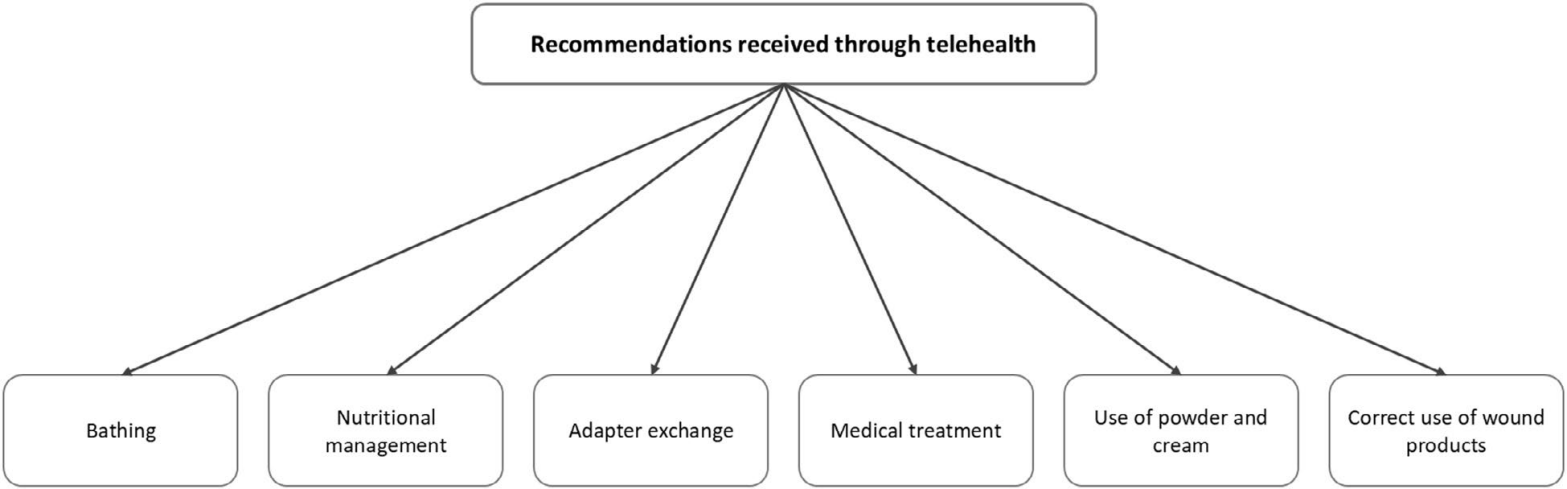
Recommendations received through telehealth, mapped across major sub‐themes including bathing, nutritional management, adapter exchange, medication use, and wound‐care products.

#### 3.2.1 Subtheme 1: Bathing

Participants frequently needed clarification on stoma‐care procedures during bathing.

I didn't know whether to take it off while bathing. You explained that I should remove it before getting into the shower, and it made cleaning much easier. (P3)

#### 3.2.2 Subtheme 2: Nutritional Management

Participants expressed a lack of knowledge about postoperative nutrition and sought clarity through telehealth.

Before the colostomy, I was very confused about what to eat. I called and got support—it was very useful. (P1)

We talked about many things, not only product management but also how and what we should eat. (P2)

#### 3.2.3 Subtheme 3: Adapter Exchange

Guidance regarding the correct materials and techniques for adapter changes helped participants manage their care more safely.

You showed me which materials to use and how to use them in detail. I'm very satisfied now. (P6)

#### 3.2.4 Subtheme 4: Medical Treatment

Telehealth also provided guidance on when to seek medical assistance and which medications were appropriate.

We had inflammation once, and you told us to see a doctor. It was a lifesaver. (P1)

They told me clearly which medication to use when I had a problem. (P6)

#### 3.2.5 Subtheme 5: Use of Powder and Cream

Participants described how correct use of powders and creams reduced irritation and improved wound healing.

We bought a zinc‐containing cream and kept the wound open longer as you recommended. (P1)

Our telenurse recommended powder and cream. As we used them, the irritation decreased over time. (P7)

#### 3.2.6 Subtheme 6: Correct Use of Wound Products

Participants valued the specific and timely feedback they received regarding wound‐care products.

I had an open wound around the stoma. You helped me understand which products to use and how. (P2)

I would open it every two days and send you pictures. You told me whether I was doing it right or wrong, and I followed that. (P5)

Summary of Theme 2:

Telehealth provided personalised and practical recommendations that addressed immediate concerns while empowering participants to manage their stoma care more confidently.

### 3.3 Theme 3: Feelings After Telehealth

Participants' emotional reflections following telehealth engagements clustered into four subthemes (Figure 4).

**FIGURE 4:**
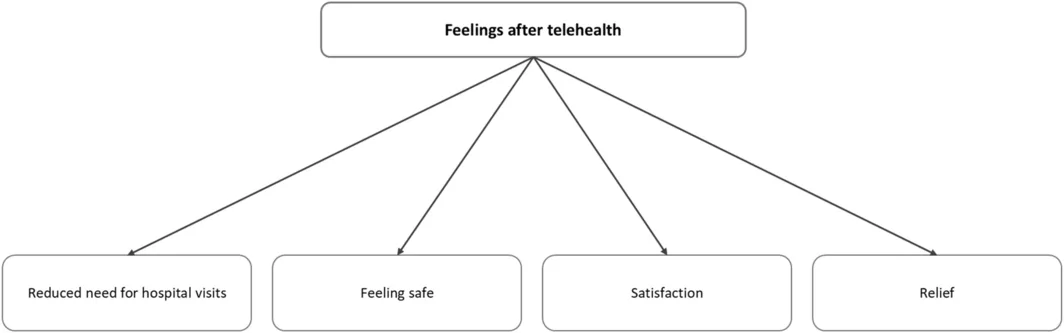
Feelings expressed by participants after receiving telehealth support, organised into four sub‐themes describing emotional and practical outcomes.

#### 3.3.1 Subtheme 1: Reduced Need for Hospital Visits

Participants frequently expressed that telehealth reduced the need for hospital‐based care, allowing them to manage most issues at home.

Without telehealth, I would have needed to go to the hospital. Getting the right information made everything easier. (P2)

#### 3.3.2 Subtheme 2: Feeling Safe

Participants described feeling safer and more secure after receiving timely guidance.

It was reassuring for me. (P7)

I felt safer in a different way. (P6)

#### 3.3.3 Subtheme 3: Satisfaction

Immediate access to professional support was cited as a major factor contributing to patient satisfaction.

Being able to reach someone immediately is a great advantage. (P1)

I'm very satisfied. (P6)

It was probably the biggest favour done to me. No one else had that technical knowledge except you. (P3)

#### 3.3.4 Subtheme 4: Relief

Telehealth support offered emotional relief, especially when participants encountered unexpected problems.

When we were stuck or worried, the support on the phone was very comforting. (P1)

Of course, it was a great relief… (P2)

Summary of Theme 3:

The emotional outcomes of telehealth—feeling safe, satisfied and relieved—demonstrate its supportive role in patients' adaptation to stoma care and product management.

### 3.4 Theme 4: Perceived Effectiveness of Counselling

Participants shared positive perceptions regarding the effectiveness of the counselling they received, expressed through three subthemes (Figure 5).

**FIGURE 5:**
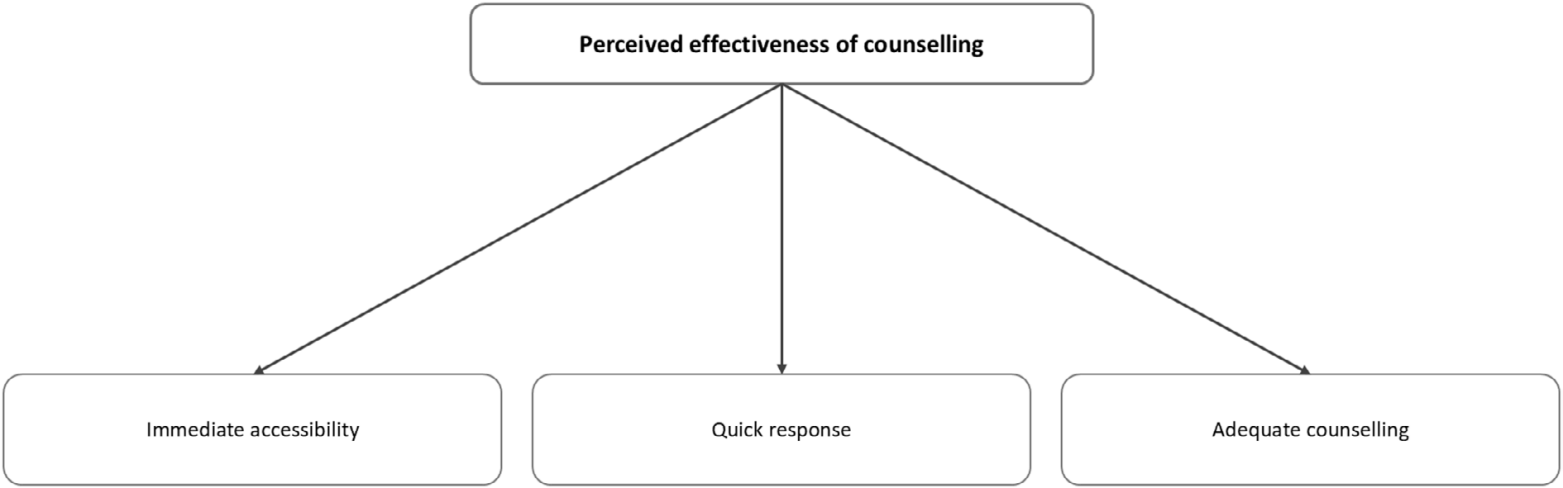
Perceived effectiveness of counselling, summarising sub‐themes related to accessibility, rapid response, and adequacy of guidance.

#### 3.4.1 Subtheme 1: Immediate Accessibility

Participants highlighted the importance of being able to reach counselling services quickly and at any time.

The counselling service is really great. I write on WhatsApp and get a response quickly—even on weekends. They took care of me immediately. (P2)

We could reach the support nurse right away. (P7)

#### 3.4.2 Subtheme 2: Quick Response

Fast responses reassured participants and enabled timely interventions.

Whenever I have the smallest problem, they respond immediately. (P4)

#### 3.4.3 Subtheme 3: Adequate Counselling

Participants perceived the telenurses' explanations as clear, detailed and technically competent.

There was sufficient attention. (P7)

No one else had such technical knowledge. The nurse explained everything clearly and supported me throughout. (P3)

Summary of Theme 4:

Telehealth counselling was viewed as accessible, responsive and sufficiently comprehensive, reinforcing its value in supporting patients' self‐management and confidence in stoma product use.

### 3.5 Theme 5: Alternative Methods

Participants also described various strategies they used outside telehealth to address their concerns (Figure 6).

**FIGURE 6:**
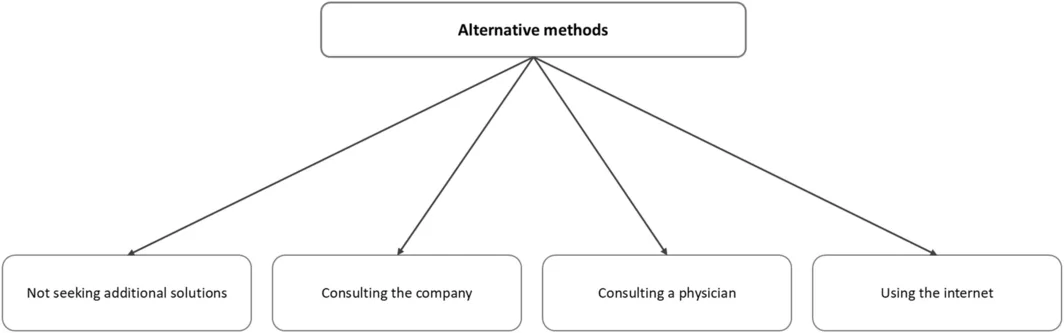
Alternative methods used by participants to address stoma‐related problems outside telehealth, including reliance on physicians, companies, internet sources, or no additional support.

#### 3.5.1 Subtheme 1: Not Seeking Additional Solutions

Some participants preferred to rely exclusively on a single trusted source of support.

I don't look for extra solutions. I deal with one person and continue with them. (P4)

#### 3.5.2 Subtheme 2: Consulting the Company

Participants often contacted the product company for additional advice.

I maintain contact with the company that provides consultancy. (P3)

#### 3.5.3 Subtheme 3: Consulting a Physician

For issues perceived as medically urgent, participants preferred consulting a physician.

I would directly ask my doctor for reliable information. (P6)

#### 3.5.4 Subtheme 4: Using the Internet

Some participants used online sources to search for information but tended to verify their findings with telehealth professionals.

I usually don't consult anyone else. I search on the internet when needed. (P2)

We check the internet, but our priority has always been our nurse. (P7)

Summary of Theme 5:

Although telehealth was a primary and trusted source of support, participants also turned to other avenues, with varying levels of trust and perceived usefulness.

## 4 Discussion

This study explored the product management experiences of individuals living with a stoma and the contribution of telehealth services to their care processes. Participants were mostly older adults who had undergone emergency surgery, predominantly due to cancer, and for most of them this was their first experience with a stoma. The majority had received stoma‐care education either from company‐based consultants or directly from stoma nurses, and all participants reported diverse experiences regarding product use and their interactions with health services (Table 1). These contextual characteristics help to frame the thematic findings of the study.

### 4.1 Reasons for Using Telehealth and Support in Managing Complications

The first main theme in this study concerned the reasons for utilising telehealth (Figure 2). Participants predominantly used telehealth services for practical issues such as adapter use, irritation management, wound care and paste applications. These findings are in line with the systematic review of Köse and Avşar (2022), which emphasised that telehealth practices enhance patient satisfaction, contribute to the rehabilitation process and play an effective role in care management. Similarly, in the present study, patients reported that telehealth provided crucial support in product selection, prevention of skin irritation and guidance during care procedures.

Consistent with the meta‐analysis by Wei‐ying et al. (2024), which showed that telehealth practices significantly reduce complication rates in patients with stoma, participants in the present study described being able to avoid recurrent problems such as leakage and irritation once they received guidance on adapter selection and skin care. Kim et al. (2023) also reported that telehealth‐supported education increases patients' knowledge levels and self‐care skills compared with traditional education methods. In parallel, participants in this study stated that they managed their care processes more effectively thanks to telehealth support. Overall, the literature and the accounts obtained in this research suggest that integrating telehealth with traditional care models may be of value for patients with a stoma.

### 4.2 Individualised Recommendations and Their Impact on Daily Life

The second main theme focused on the recommendations provided through telenursing, including bathing, nutritional management, adapter change, medical treatment, use of powder and cream and correct use of wound products (Figure 3). Participants reported that the recommendations they received via telehealth facilitated their care processes and, in their view, improved their quality of life. Akarsu et al. (2023) demonstrated that telehealth is effective in supporting post‐discharge self‐care in patients who have undergone trauma‐related lower‐extremity fracture surgery. Similarly, in this study, telehealth facilitated patients' adaptation to daily activities such as bathing, changing adapters and caring for wounds. Participants described managing hygiene processes more consciously and performing adapter changes with greater confidence following telehealth support.

The study by Sevinç and Aktürk (2024) emphasised that evidence‐based telehealth practices improve the quality of healthcare services by increasing access to counselling, care management and multidisciplinary services. The present findings likewise indicate that participants perceived telehealth as a source of accurate information on nutritional management, which they described as helping them avoid problems and maintain control over bowel movements. Akpınar Söylemez and Özgül (2022) reported that telehealth facilitates access to health services for older adults, reduces hospitalisation rates and supports individuals in becoming more independent in their care processes. In parallel, participants in this study expressed that telehealth services increased their self‐care skills and helped them manage product use and skin care more consciously. Specifically, participants described fewer episodes of skin irritation after using powders and creams more judiciously, based on telenurses' recommendations. Zewude et al. (2021) noted that the quality of life of patients with stoma is affected by multiple factors and that nutritional management, depression and social isolation pose significant challenges. The findings of the present study indicate that participants viewed telehealth support as guiding them in addressing some of these problems, and they associated more appropriate wound‐care product use with fewer skin and wound complaints and with a better perceived quality of life.

The third main theme concerned patients' experiences after receiving telehealth services, including a decreased need to visit the hospital, an enhanced sense of security, satisfaction and feelings of relief (Figure 4). Participants indicated that, in their experience, telehealth contributed positively to both their physical and psychological well‐being. Mercan Annak et al. (2021) showed that telehealth is effective in reducing hospital visits by ensuring continuity of patient care. In a similar way, patients in this study reported a reduced need to visit the hospital thanks to telehealth support. Being able to resolve problems remotely through professional guidance was described as a significant advantage.

Brady et al. (2025) emphasised that telehealth‐supported digital systems enhance patient safety and increase patients' sense of security during care processes. In the present study, patients stated that telehealth services instilled confidence and reduced their feelings of isolation when they encountered problems. Participants, particularly those recently diagnosed, described feeling less anxious and better able to manage their care processes. The findings of this study are consistent with the literature, indicating that the presence of a reachable professional makes patients feel safer and more supported.

In this study, patients reported that increased access to healthcare professionals through telehealth created a significant sense of relief. Oran and Yurt (2023) examined the effects of telehealth on patient psychology and found that telehealth reduces patients' anxiety levels and provides psychological relief. In our study, participants stated that the support they received from telenurses regarding their concerns was relieving and that the elimination of uncertainties due to lack of information contributed to a more stable psychological state.

The fourth main theme concerned the effectiveness of counselling and encompassed patients' experiences of the telehealth counselling services they received (Figure 5). Participants reported that the support they obtained from telenurses was timely and effective, and that the service offered positive experiences in terms of speed, accessibility and professional competence. Immediate access to telehealth counselling was perceived as a critical factor in the effectiveness of the process. In a study on surgical patients' access to telehealth services, it was reported that telehealth allowed individuals to receive counselling without visiting the hospital, thereby increasing satisfaction (Özkan and Asar 2022). In line with this, participants in the present study stated that they could receive support from telenurses even on weekends and that this continuity gave them confidence.

Providing rapid responses to patients is one of the most important advantages of telehealth. In a study examining telehealth use in high‐risk groups during the COVID‐19 pandemic, remote services were reported to be critical, particularly for individuals with limited access to hospitals, and to reduce patients' stress levels (Arpag et al. 2022). According to the results of the present study, participants similarly indicated that rapid feedback from telenurses enabled them to address health problems earlier. Adequate telehealth counselling also helps patients receive psychological support and manage their care processes more effectively. A study examining the use of telehealth in home care observed that information support provided to patients to better manage their care processes increased their self‐care capacity (Ersoy et al. 2015). In this study, participants reported that the information provided by telenurses was detailed, accurate and comprehensible and that they could obtain additional explanations when necessary.

In a meta‐analysis conducted in Turkey, it was demonstrated that the quality of information received from health services has a direct impact on self‐care capacity and quality of life (Fidan 2024). Consistent with this, participants in the current study stated that the counselling they received from telenurses not only addressed emergencies but also contributed to long‐term care management and was sufficient in meeting their informational needs.

The fifth main theme addressed alternative methods and focused on patients' tendencies to seek additional solutions beyond telehealth (Figure 6). Although telehealth provided a reliable support mechanism for many participants, some preferred to obtain information and support from other sources. Patients indicated that, while they were satisfied with telehealth services, in certain situations they perceived physicians' opinions as more reliable, especially in the presence of unexpected complications. Başoğlu and Polat (2023) reported that telehealth applications facilitate access to health information but that individuals may still turn to alternative sources, such as the internet, in some cases. Consistent with these findings, participants in this study reported that they searched for health information on the internet, but they frequently consulted telehealth professionals to verify the accuracy of that information. Patients acknowledged that the internet offers rapid access to information; however, they did not consider it sufficient as a stand‐alone source when making health‐related decisions. These results suggest that telehealth functions as a central, trusted reference point, even when patients explore other information channels.

### 4.3 Limitations and Structural Challenges in Telehealth Use

Although these findings highlight the potential benefits of telehealth practices, some limitations must be considered for telehealth to be effective on a larger scale. One of the most important challenges is the difficulty in establishing empathic rapport due to the lack of face‐to‐face communication, which may be particularly disadvantageous for patients requiring emotional support (Keskin and Özhelvacı 2022). Internet connectivity issues and data security concerns can interrupt services and pose risks to patient confidentiality (Rettinger and Kuhn 2023). In patients with a stoma, limited access to and familiarity with digital tools, particularly among older individuals, may restrict the extent to which such services can be used (Soares‐Pinto et al. 2023). Furthermore, in Turkey, inadequate legal regulations pertaining to telehealth practices restrict the standardisation and effectiveness of these services (Keskin and Özhelvacı 2022).

Taking these limitations into account, the findings of this study suggest that telehealth has considerable potential to support patients with a stoma in managing product use and skin care, but that this potential can only be fully realised if technological, educational and legal infrastructures are strengthened.

## 5 Limitations

This study has several limitations that should be considered when interpreting the findings. First, all participants were recruited from a single private healthcare provider offering telenursing services, which limits the geographical and institutional diversity of the sample. Although qualitative research does not aim for statistical generalisation, this sampling structure may restrict the transferability of the findings to other settings or patient populations. Future studies could include participants from different regions and care organisations to broaden the contextual scope.

Second, the data were based on participants' retrospective accounts of their telehealth experiences. Such accounts may be influenced by recall bias, as individuals reconstruct past experiences in light of current emotions and adaptation processes. Nevertheless, such retrospective accounts remain valuable in qualitative descriptive research, as they reflect how participants interpret and make sense of their care experiences.

Third, the telenursing service examined in this study was delivered as part of a broader package of care rather than as an isolated intervention. In the absence of a comparison group, the observed benefits cannot be attributed specifically to the telenursing service itself; therefore, the findings should be interpreted as reflecting participants' experiences of telehealth within this wider context of care.

Fourth, the interviews were conducted by a member of the research team who had previously provided telenursing support to the participants. Although the measures described in Section 2.4 were taken to reduce social desirability bias, participants may nonetheless have been inclined to describe the service favourably, and this should be taken into account when interpreting the predominantly positive accounts reported here.

Finally, the coding and preliminary analysis were conducted by a limited number of researchers, which may introduce interpretive bias. To enhance rigour, the analysis followed a systematic coding process; peer review was conducted by another qualitative researcher and direct quotations were used to support the credibility of the themes. Member checking was utilised in this study; however, further methodological triangulation in future research may strengthen the trustworthiness of findings.

## 6 Implications for Nursing Practice

The findings offer several directions for stoma nurses and telenurses. Participants' accounts of leakage, poor adapter fit and peristomal irritation indicate that product‐use education should be revisited after discharge rather than delivered only in the immediate postoperative period, and that it should address the selection and correct application of adapters, pastes, powders and creams. Leakage management warrants particular attention, as participants described recurrent leakage as the trigger for most of their contacts; the guidance they received typically combined a review of adapter type and fit with advice on the use of paste and barrier products, which suggests that a structured leakage assessment sequence would be a useful addition to remote follow‐up. Routine skin assessment can be supported remotely, as participants frequently described sending photographs of the peristomal area and receiving guidance in return; services adopting this practice require clear protocols for image quality, secure transmission and documentation. Structured, scheduled follow‐up contacts may be more useful than reactive contact alone, since participants valued the availability of a known professional, including at weekends. Clear escalation criteria are also needed, as participants described being advised to consult a physician when signs of infection appeared; telenursing protocols should therefore specify the clinical thresholds at which remote guidance is replaced by referral. More broadly, the exchange of photographs and written advice described by participants indicates a need for explicit digital communication protocols covering consent, image quality, secure transmission, record‐keeping and expected response times. Finally, services should anticipate that patients consult additional sources, particularly the internet, and should position telenursing as a point at which such information can be verified.

## 7 Conclusion and Recommendations

This study examined the product management experiences of individuals living with a stoma and receiving telehealth support. The findings show that patients encounter a range of challenges—such as adapter selection, skin irritation, wound care and nutritional management—and that they experienced telehealth as providing timely, accurate and reassuring guidance that facilitated daily care. Participants described an enhanced sense of safety, a reduced need for hospital visits and greater confidence in their own self‐care.

These results highlight the importance of integrating structured telehealth services into routine stoma care. Ensuring that nurses are prepared to deliver evidence‐based digital care may improve continuity of care and support patients' adaptation following stoma surgery.

Future research should investigate the long‐term impact of telehealth on complication rates, quality of life and sustained self‐care behaviours, and explore ways to optimise digital tools and address technological barriers in diverse patient populations.

## Author Contributions

**Gökçe Akkaya:** data curation, resources, writing – review and editing, writing – original draft, funding acquisition. **Seçil Taylan:** supervision, writing – review and editing. **Fatma Eti Aslan:** supervision, writing – review and editing. **Kübra Can:** investigation, funding acquisition, writing – review and editing. **Evin Korkmaz:** investigation, methodology, validation, writing – review and editing, writing – original draft, supervision, project administration, formal analysis.

## Funding

The authors have nothing to report.

## Ethics Statement

Ethical approval for this study was obtained from the Bahçeşehir University Scientific Research and Publication Ethics Committee prior to data collection (Approval Date: 01.08.2024, Meeting No: 2024/07). All procedures performed in this study were conducted in accordance with the ethical standards of the institutional and national research committee and with the principles of the Declaration of Helsinki.

## Consent

Written informed consent was obtained from all participants before conducting the interviews. Participation was voluntary, and participants were informed that they could withdraw at any time without any consequences.

## Conflicts of Interest

G.A. works as a consultant telenurse for the private healthcare provider whose stoma‐care telenursing service was the focus of this study and conducted the participant interviews. The remaining authors declare no competing interests.

## Data Availability

The data that support the findings of this study are available from the corresponding author upon reasonable request. The data are not publicly available because they contain information that could compromise the privacy of the research participants.
